# Noninvasive Ventilation in Palliative Care and Ethical Dilemma

**DOI:** 10.3389/fped.2020.00483

**Published:** 2020-08-27

**Authors:** Uros Krivec, Serena Caggiano

**Affiliations:** ^1^Department of Pediatric Pulmology, University Children's Hospital Ljubljana, University Medical Center Ljubljana, Ljubljana, Slovenia; ^2^Laboratory Pediatric Pulmonology and Respiratory Intermediate Care Unit, Sleep and Long Term Ventilation Unit, Academic Department of Pediatrics (DPUO), Pediatric Hospital “Bambino Gesù” Research Institute, Rome, Italy

**Keywords:** noninvasive ventilation, pediatric, palliative care, ethics, decision-making

## Abstract

Significant difference exists between validated indications for noninvasive ventilation (NIV) use in children and current real life practice. Lately, dedicated centers have reported exponential growth of NIV use in children and adolescents. Upper airway obstruction, neuromuscular diseases, chronic lung/thoracic conditions, and central respiratory drive failure remain the most prevalent indications. However, the need to alleviate respiratory failure related distress has been increasingly recognized in several other conditions. Palliative care in children with life limiting disorders is a complex continuum of activities. In order to provide the most appropriate care for the patients and their families, the management often oscillates between actively curative and purely supportive actions. Despite unprecedented therapeutic advancements, several neurologic, metabolic, hemato-oncologic, respiratory, and other rare diseases remain with no curative options. Besides, attentiveness to relive suffering, awareness, and availability have moved the boundaries of NIV use toward conditions formerly not considered suitable for such care. Still, NIV has limitations and can, if sustained in inappropriate circumstances, fail to provide relief. A structured professional frameshift should be available for support and ethical guidance in order to provide confidence to patients, families and all the involved caregivers.

## Introduction

Over the last decades, noninvasive ventilation (NIV) has become an established treatment modality in children. Technical advancements and availability of suitable interfaces have expanded its use in patients with diverse medical conditions. Long term NIV has been shown to positively influence many important disease outcomes. However, a large gap still exists between proven benefits and real-life use of this rapidly evolving respiratory support (1).

Many severe pediatric conditions have been linked to long term NIV, notably the neuromuscular diseases (NMD). A lot of such illnesses still lack causative treatments. Thus, appropriate respiratory care represents an important part of patients' management throughout the entire disease course.

The need for dedicated palliative care has been recognized in these children. Pediatric palliative management often spans over long time periods. Several specific aspects have been identified in these circumstances, particularly the fluctuating balance between adequate measures of active treatment and palliative support (2).

## Noninvasive Ventilation

The main goal of NIV is to appropriately sustain the ability of the respiratory system to meet the body's metabolic demands. Studies confirm that NIV can adequately relieve dynamic obstruction in the large airways, support the respiratory pump function in NMD and chest abnormalities, alleviate breathing in patients with parenchymal lung disorders and back-up diseased central breathing control (3).

An important advantage of NIV is its time-limited activity and respect for the patient's body integrity. Patients may need support only during certain activities or on demand like when sleeping, speaking or eating. Such respiratory aid is possible through a removable interface and does not interfere with person's life when breathing assistance is not essentially needed.

NIV has a clear survival benefit in many severe progressive illnesses, especially in NMD like spinal muscular atrophy (SMA) and Duchenne muscular dystrophy (DMD) (4, 5).

## Pediatric Palliative Care

Pediatric palliative care aims to appropriately relive symptoms, sustain quality of life and support the whole family of the affected child. Provided activities should be oriented toward reliving physical, psychological, social, and spiritual suffering in patients and their family members (6).

The Guide to Children's Palliative Care (2) comprehensively defines four categories of patients with life-limiting and life-threatening conditions also known as the “Together for Short Lives” (TfSL) pediatric palliative care categories. Category 1 includes diseases where curative treatment may be feasible but can also fail. These are predominantly children with hemato-oncological conditions and congenital heart disease. Category 2 contains conditions where premature death is inevitable, but available treatment can importantly prolong life and its' quality. Several NMD and congenital metabolic conditions have lately moved to this group due to therapeutic advancements. Category 3 lists progressive conditions without curative treatment options. Provided activities cannot stop disease progression and mainly aim to improve the quality of life. A lot of neurologic and metabolic conditions still remain in this category. Category 4 consists of irreversible but non-progressive conditions causing severe disability leading to susceptibility to health complications and likelihood of premature death. These are patients that suffer from injury related central nervous system damage.

In contrast to the general beliefs that palliative care predominantly concerns end-stage cancer patients, conditions in categories 2–4 affect the large majority of children in need of palliative management nowadays. Only a dedicated multidisciplinary palliative team can offer optimal support to the affected children and their families (7).

Relief of dyspnea and strenuous breathing represent the key elements of palliative care. Hunger for air is one of the most frightening symptoms for children and their parents. It is often reported to cause considerable suffering of the affected patients and even prolong parental grief (8).

Management of dyspnea in children with advanced respiratory conditions remains challenging. Several possibilities should be considered. It is important to maintain good airway hygiene. Sensation of an air draft to the face (like the use of hand held fen) and other nonpharmacological activities can reduce anxiety in patients and caregivers. A number of pharmacological options are also available; especially opioids, anxiolytics, and diuretics. Besides, providing supplemental oxygen and NIV can be beneficial (9).

## Noninvasive Ventilation in Pediatric Palliative Care

Only a modest number of publications report on the use of NIV for palliative purposes in children even though this respiratory support modality is increasingly used in numerous pediatric life-limiting diseases.

Clinicians consider NIV as an important tool in palliative care aimed at providing comfort to patients, relieve suffering, and improve quality of life of both children and their families. A contemporary survey on the use of NIV in pediatric palliative care (10) gathered responses from 73 participants working at University hospitals. Sixty-two percent were intensivists, a quarter pediatric pulmologists and 11% palliative medicine consultants. Large majority (84%) considered the use of NIV as an appropriate measure to relief dyspnea due to acute respiratory failure in children with a “do-not-intubate” (DNI) order. A lower proportion, about two thirds of responders, considered NIV appropriate in DNI children with progressive respiratory failure. They considered severity of dyspnea to be the most important indicator to start NIV. The efficacy of provided care was evaluated by clinical measures, the level of attained comfort, and child's and family's satisfaction. Monitoring of gas exchange parameters was only rarely used. Six percent of participants included NIV in their advanced care planning.

In certain instances, decisions on mechanical respiratory support determine the expected disease outcome. The level and modality of provided respiratory support can ultimately preclude the question of life or death. Chatwin et al. (11) reported offering NIV and mechanical insufflation/exsufflation to a group of 13 children with SMA type 1. All the treatment options were discussed with the families. In infants younger than 3 months, NIV was offered primarily as a palliative measure. It was seen that NIV could allow for a successful extubation, eased respiratory symptoms, altered progression of chest deformations and facilitated transfer to the home. The latter was particularly valued by the parents. The authors stressed the importance of the goal-directed approach in palliative NIV for patients with incurable conditions.

Several new therapeutic options have recently reshaped the management of patients with SMA. These possibilities also altered the purpose of NIV in this group of patients. A recent multicenter study addressed the aspects of palliative care in SMA 1 children through parents' reports (12). In the 5 year, observational period (2012–2016), 80 patients from 17 different centers were included, partly in a prospective manner (46%). In the observed period, seven patients started receiving specific treatment (nusinersen). As expected, survival of patients in the nusinersen group was significantly longer than in non-treated group (57 vs. 1%, *p* = 0.001). Four out of seven patients on nusinersen (57%) were also supported by home NIV, compared to only 8% in the group without specific treatment (*p* = 0.006). Data on care at the time of death were available for the prospectively followed patients only. During the last 48 h before death, 2 children (5%) received NIV. Sedation and analgesia was given to 81% of patients. Pediatric palliative care team was involved in 74% of cases. This article emphasized the importance of the proactive pediatric palliative management in SMA 1 children, the shift of specialized care to the home and consequently even more intensive involvement of the parents.

Palliative NIV is a precious treatment aimed to alleviate berating and thus improve the quality of life. Besides its use in children with NMD, reports indicate positive results in other conditions, too. Bosch-Alcaraz (13) reported on short-term effects of NIV use in 55 pediatric patients managed at a single palliative unit over 7 consecutive months. Large majority of children suffered from NMD (80%), followed by oncological, cardiac, and respiratory illnesses. Silverman Anderson scale was used to grade dyspnea and Edmonton symptom assessment system to evaluate comfort and pain. Over the 24 h observational period dyspnea and pain levels improved in all patients under NIV. Besides, rise in SpO_2_/FiO_2_ ratio, decrease in heart and respiratory rates were documented. Only minor NIV related complications were noted. NIV provided amelioration and improved comfort in all treated children.

The use of NIV for palliative purposes varies considerably in different societies. Incurable and progressive or static and frail illness often lead to respiratory insufficiency in advanced stages. Girbal et al. (14) retrospectively reviewed characteristics of children with complex obstructive sleep apnea treated with either CPAP or NIV over a 15 year period. Data from 68 pediatric and adolescent patients were analyzed. Participants suffered from congenital malformations/genetic disorders (50%), cerebral palsy (13%), central nervous system tumors (12%), inborn errors of metabolism (9%), and other conditions. Authors reported clinical improvement in 53 compliant children. Improvement in sleep disordered breathing parameters was formally recorded in 29 patients. Although the provided respiratory support could be regarded as a palliative treatment measure in several described children and adolescents, researchers specifically indicated palliative NIV use to improve comfort and decrease hospital stay in four cancer patients.

Optimal care of complex patients requires a holistic consideration of expectations and available management options. A retrospective analysis of 198 children, adolescents, and young adults treated over 32 months at a single specialized pediatric palliative care center (15) revealed high symptom burden in all patient. Distinct symptom clusters were linked to patients in specific TfSL categories (2). Dyspnea was reported as a troublesome symptom in all patient groups except for those in category 1. Overall, patients in category 4 suffered the most pronounced symptoms, especially related to the neurological and respiratory domain. Among the available care tools, presence of a ventilator was reported in up to 30% of patients in category 2.

Nolte-Buchholtz et al. (16) described characteristics of newly referred patients to nine tertiary specialized pediatric palliative home care teams over a period of 6 months. Seventy-five patients with a median age of 7.7 years (range 0–31 years) were included. Twenty-one (28%) were treated for cancer. In the non-cancer group, majority of patients suffered from NMD (52%), followed by neurodegenerative conditions (17%), chromosomal and cardiovascular diseases (11% each). Only one patient had a primarily respiratory disease. In both groups, counseling and symptoms management were offered in over 85% of cases. Overall, compromised communication, pain, swallowing difficulties, and cognitive impairment represented the most troublesome symptoms. Dyspnea was significantly more often expressed by non-oncological patients (50 vs. 19%, *p* = 0.019). Ventilatory support was needed in non-cancer group only; five patients (9%) required NIV, six patients (11%) were ventilated trough tracheostomy. The authors emphasized the medical complexity of the enrolled patients and consequently the need for a committed multidisciplinary palliative care team.

Chatwin et al. (17) analyzed the characteristics of patients who died while receiving long term noninvasive respiratory support at a single large-volume tertiary center. Over an 18 year period, the authors report 109 deaths in 449 patients treated with either NIV or continuous positive airway pressure (CPAP). Seventy-six percent of those who died suffered from NMD. This work was not primarily oriented toward the evaluation of palliative care, but some detailed data were presented for 55 patients. Ten died at the center, palliative extubation was mentioned in one case. Five patents died from expected respiratory failure while on palliative care; four at home, and one in the hospice. The authors specifically stressed the importance of providing palliative care over long time periods with oscillations between active interventions for the management of reversible conditions and times with purely supportive measures.

During the end-of-life phase NIV is reported to improve quality and extent of life. Caregivers perceived improvements in comfort and anxiety relief in patients using NIV at the end of life. Furthermore, this treatment reduced hospital stay. During the last days of life, patients sometimes choose to discontinue NIV or to limit its use and favor the ability to communicate, eat or engage other activities that they consider important (18).

Tolerability may be limited by mask discomfort, discomfort from the air pressure, claustrophobia, poorly managed initial set-up, and child or parent anxiety. The use of NIV can't be designed only to prolong the dying process with no improvement, or possible detriment, to the child's symptom burden and quality of life. Optimal symptom management should relieve suffering and thereby sustain the perceptions of decency in patients and their families during the terminal care.

Presented studies exposed a vast diversity and considerable medical complexity of pediatric patients receiving palliative NIV. Mainly observational evidence supports its ability to reduce suffering and provide comfort. Nevertheless, a voice of consciousness warns us that mere technicalia should not obscure the principle needs of patients and families coping with unforgiving diseases (19).

Family members have to be involved in palliative NIV decision-making process. They often provide the required every day care and therefore understand the patient's needs at best. Family should be able to dynamically modify therapeutic goals according to the clinical evolution. Clinicians have to respectively consider patients' and families' wishes primarily oriented toward quality of life and dignity (20).

Management of dyspnea is challenging. Several palliative strategies need to be considered in order to attain relief. Patients with dyspnoea can benefit from simple measures to enhance the sensation of air draft. Besides, provision of supplemental oxygen in the cooled airflow further improved the perceived hunger for air (21).

Opioids remain the mainstay to reduce symptomatic dyspnoea and to manage pain. In addition, non-opioid drugs, including local anesthetics, steroids, nonsteroidal anti-inflammatory drugs, acetaminophen, ketamine, and substances with anti—neuropathic action, can be considered. Benzodiazepines are used for sedation of patients with terminal delirium. This condition requires careful assessment. Attention should be paid to known contributing factors like alterations in sleep and other circadian activities (22).

Diuretics significantly help children with fluid retention and dyspnoea associated with pulmonary oedema (9).

## Ethical Dilemma

Epidemiological data on long term NIV use in children have identified patient groups that are commonly treated with this respiratory support modality but also point to certain differences. While upper airway obstruction, NMD and pulmonary/chest disorders represent common conditions where NIV is provided, certain reports indicate not infrequent use also in children with cerebral palsy, neurodegenerative disorders, and rare syndromic illnesses (15, 23, 24). Such real-life findings reflect important differences in the management attitudes throughout different societies.

Still, majority of children on long term respiratory support reported leading rich lives. To the contrary, almost one quarter of the caregivers graded the burden of care as severe. Over a half of them suffered from chronic illnesses including anxiety and depression. Providing for a child with such a complex medical condition not infrequently led to family disruptions, single parenting and negative influences on other siblings' lives (25, 26).

Ethical considerations also concern the awareness of the intrinsic level inequality between the patient and family in need of help and the medical team or institution in possession of the means to provide help. Shared decision making should address all positive and negative aspects of the patient's current life condition with special concern to the expected disease course in the future (27).

Technical advancements have made long term respiratory support possible in children with severe chronic health conditions. Moreover, novel therapies and emerging prospects for further expansions in the near future can importantly influence decisions on long term management. Alongside these possibilities it should be recognized that unrealistic expectations might prolong unnecessary suffering and even lead to futile interventions in an individual child.

An ethical framework for decision making in pediatric long term ventilation has been proposed (28). This approach can provide appropriate space for the interaction between the involved patients, their families, healthcare providers, health authorities and the broader society. We believe the patients and families should have the central role in the process, but they need realistic information and appropriate guidance from a dedicated multidisciplinary team (29). The framework should also provide directions on further steps, when consensual decisions could not be reached.

## Future Directions and Research

Current overview exposed paucity of studies addressing the use of NIV in pediatric palliative care. Further research is needed in order to identify benefits and limitations of this treatment modality throughout the vast domain of palliation in children, since NIV has been advocated in the care of various diseases and conditions spanning over long time periods, decisively beyond the narrow frame of terminality. Collaborative multicenter study design, standardized objective evaluations and individual centered research methods should be used to overcome the small number of patients at individual centers, divers, and often descriptive outcomes and single patient and family oriented interventions.

Shifts of established boundaries have exposed important ethical dilemma. An appropriate forum for scientific and public discussion should be made available in order to express doubts, stimulate broad reasoning and thus promote actions in the best interest of all involved.

## Conclusions

NIV should be considered as one of possible options to attain breathing relief in children with either imminent or long lasting life-limiting conditions. However, decisions on any palliative action must follow patients' and families' preferences, perceptions and the relevance of time. A structured professional frameshift should be available for support and ethical guidance in order to provide confidence to patients, families, and all the involved caregivers.

## Author Contributions

UK drafted, revised, and approved the manuscript. SC revised and approved the manuscript. All authors contributed to the article and approved the submitted version.

## Conflict of Interest

The authors declare that the research was conducted in the absence of any commercial or financial relationships that could be construed as a potential conflict of interest.
